# Gene abnormalities in primary congenital glaucoma in Saudi Arabia: a study of known genes and search for novel genes

**DOI:** 10.1186/1471-2164-15-S2-P33

**Published:** 2014-04-02

**Authors:** Osama M Badeeb

**Affiliations:** 1 Ophthalmology, King Abdulaziz University, Jeddah, Saudi Arabia

## Background

Primary congenital glaucoma (PCG) refers to patients with isolated anomaly of the trabecular meshwork and glaucoma onset within the first 3 years of life [1]. It is relatively common in Saudi Arabia, with estimated incidence of 1: 2500 live birth, due to high prevalence of consanguinity within Saudi's families [2]. CYP1B1 is the most commonly mutated gene in PCG. This study was undertaken to identify mutations in CYP1B1 and looking for novel genes, by screening blood of patients who had typical findings of PCG, with high-resolution SNP microarray, homozygous mapping and direct sequencing of CYP1B1.

## Results

34 patients were studied; 18 patients belonged to 9 families, and 16 patients were non-familial, isolated PCG. Consanguinity was present in 28/34 (82.4%) of cases. All patients were diagnosed to have bilateral PCG at birth except one child. Male (21) > female (13). 82.4% (28/34) of patients were solved with pathogenic mutations and (6/34)17.6% remained unsolved. Direct sequencing of exon 2 revealed two pathogenic variants (p.Gly61Glu, p.Glu229Lys). P.Gly61Glu substitution was found both homozygous in 60.7% (17/28) and heterozygous in 3.6% (1/28) patients. P.Glu229Lys variant found heterozygous in 3.6% (1/28) cases. Two pathogenic variants (p.Ala443Gly, p.Arg469Trp) were found in exon 3. P.Ala443Gly was present in 3.6% (1/28) cases, while p.Arg469Trp was found in 28.6% (8/28) patients. All variants had been reported previously in Saudi population, except p.Glu229Lys.

## Conclusions

P.Gly61Glu mutation is frequently present in PCG in Saudi Arabia and p.Glu229Lys is a novel one.
